# Fewer rainy days and more extreme rainfall by the end of the century in Southern Africa

**DOI:** 10.1038/srep46466

**Published:** 2017-04-13

**Authors:** Benjamin Pohl, Clémence Macron, Paul-Arthur Monerie

**Affiliations:** 1 Centre de Recherches de Climatologie, UMR6282 Biogéosciences, CNRS/université de Bourgogne Franche Comté, Dijon, France; 2 National Center for Atmospheric Science, Department of Meteorology, University of Reading, Reading RG6 6BB, UK

## Abstract

Future changes in the structure of daily rainfall, especially the number of rainy days and the intensity of extreme events, are likely to induce major impacts on rain-fed agriculture in the tropics. In Africa this issue is of primary importance, but the agreement between climate models to simulate such descriptors of rainfall is generally poor. Here, we show that the climate models used for the fifth assessment report of IPCC simulate a marked decrease in the number of rainy days, together with a strong increase in the rainfall amounts during the 1% wettest days, by the end of the 21st century over Southern Africa. These combined changes lead to an apparent stability of seasonal totals, but are likely to alter the quality of the rainy season. These evolutions are due to the superposition of slowly-changing moisture fluxes, mainly supported by increased hygrometric capacity associated with global warming, and unchanged short-term atmospheric configurations in which extreme events are embedded. This could cause enhanced floods or droughts, stronger soil erosion and nutriment loss, questioning the sustainability of food security for the 300 million people currently living in Africa south of the Equator.

The long-term evolution of tropical rainfall is often uncertain, current climate models having difficulties for simulating the spatial and temporal variability of atmospheric convection. As a result, the consensus between climate models is generally much weaker for rainfall than for temperature changes. Most parts of Southern Africa experience their main rainy season in austral summer, from November through February, in line with the southernmost location of the Inter-Tropical Convergence Zone at this time of the year. In the future decades, seasonal rainfall totals simulated by the so-called CMIP5 models (Coupled Model Inter-comparison Project phase 5) exhibit weak to moderate changes compared to the current situation (shown in Fig. 1a), that mostly consist of wetter conditions over tropical Africa and contrasted changes further south in the subtropical areas (Fig. 1d–g). This mean picture conceals sizeable differences from one model to another, and the number of climate models in agreement with the multi-model averaged evolution remains weak (typically 5–10 out of the 15 used in this work: see Supplementary Table 1 for further details). One noticeable exception is Equatorial East Africa, the tendency of the “Short Rains” towards more humid conditions being a robust feature of climate change in Africa^1^.

Deeper changes are obtained with the number of simulated rainy days (Fig. 1h–k, to be compared to Fig. 1b), generally decreasing over the continent, especially at the tropical and subtropical latitudes^2^,^3^. The consensus between the models is rather high (reproduced by at least 75–80% of the models) over southern Tanzania, northern Mozambique and the neighboring of Lake Malawi. The amplitude of these changes is much weaker in the optimistic (RCP2.6) greenhouse gas emission scenario than in the pessimistic one (RCP8.5: Fig. 1j,k). The decrease is particularly strong and consensual in the southern mid-latitudes between 40 and 50°S. It is due to the southern shift of the storm track and jet streams, leading to a weakening the “roaring forties” but enhancing the “furious fifties”. This can be seen as an effect of the increasing human influence on the climate throughout the century; moreover, during the first half of the century, this shift is also partly counter-balanced by the opposite influence of stratospheric ozone recovery^4^,^5^,^6^,^7^. Over the tropical oceans, opposite-sign changes prevail, over both the Atlantic and Indian basins, contributing to the wetter conditions projected over the region at the low latitudes (Fig. 1d–g). Future modifications in the mean intensity of rainy days are of smaller amplitude (Fig. 1l–o): over Southern Africa they lead to slightly weaker amounts in the tropical latitudes and the reverse in the subtropical areas. Except in the mid-latitudes, results are strongly model-dependent, as it is often the case with tropical rainfall.

## A robust intensification of extreme rainfall events

**Figure 1:**
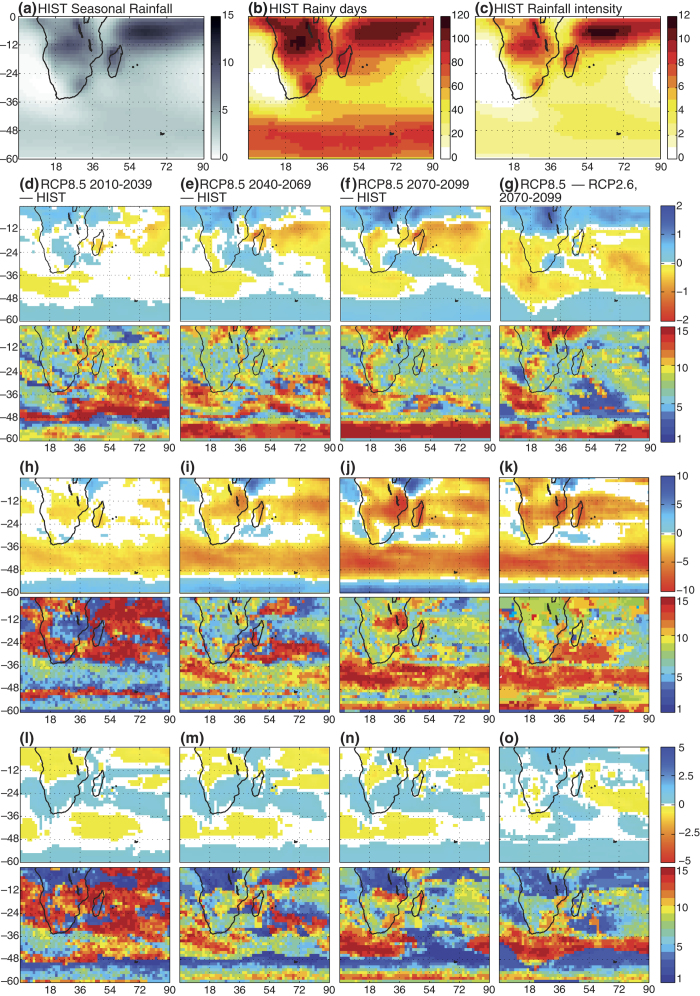
Evolution of Southern African summer rainfall. (**a**–**d**) Multi-model seasonal mean rainfall differences (mm day^−1^) against historical runs (period NDJF 1970–1999) for RCP8.5: 2010–2039 (**a**), 2040–2069 (**b**), 2070–2099 (**c**), and RCP8.5 minus RCP2.6 for period 2070–2099 (**d**). Only significant differences at the 95% level are represented. Lower panels show the number of models (out of 15) that are in agreement (changes of the same sign, with the same statistical significance level) with the above panels. (**e**–**h**) As (**a**–**d**) but for the number of rainy days (≥1 mm day^−1^). (**i**–**l**) As (**a**–**d**) but for the average intensity of rainy days (mm day^−1^).

**Figure 2:**
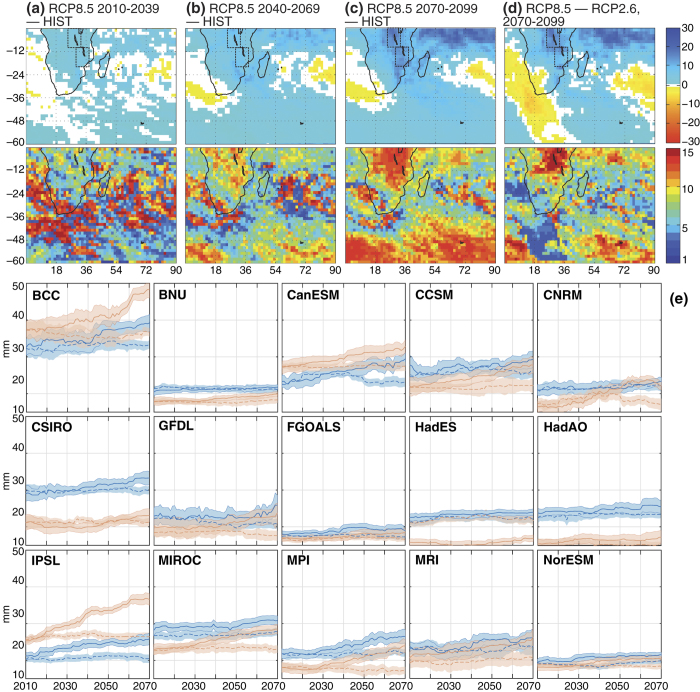
Evolution of the intensity of rainfall extreme events. (**a**–**d**) As Fig. 1(a–d) but for the 99^th^ percentile of daily rainfall amounts. Boxes represent the Tanganyika (0–11°S, 25–34°E) and Malawi (10–20°S, 30–38°E) regional indices. (**e**) Evolution of the 99^th^ percentile of daily rainfall amounts averaged over the Malawi (blue) and Tanganyika (red) regions, computed over a 29-year long moving window, and for RCP8.5 (solid curves) and RCP2.6 (dashed curves) and for the 15 climate models retained in this study. Sampling uncertainties are quantified through 1,000 bootstrap data samples: the thick curves show the median p99 values and the error bounds correspond to ±2 standard deviations. The years in the x-axis correspond to the first years of the moving windows.

Much more robust evolutions concern the tail of the distribution, that is, the intensity of extreme rainfall events. Figure 2 shows the changes in the 99^th^ percentile (p99) of daily rainfall amounts, i.e. the minimum amounts associated with the 1% wettest days. Similar results are found with the 90^th^ or 95^th^ percentiles (not shown). Future decades should be characterized by a strong increase in the p99 value (Fig. 2a–c), very robust (at least 80% of the models) in the tropical latitudes^2^,^8^,^9^,^10^,^11^. Similar evolutions can also be found in reanalyses over the recent decades (Supplemantary Figure 2), even though their reliability concerning rainfall variability, and more particularly extreme rainfall, remains questionable. In the future, they could be significantly mitigated by reduced anthropogenic emissions of greenhouse gases (Fig. 2d). Together with the decrease in the number of rainy days, these results imply that the contribution of intense events to the seasonal amounts should sensibly increase over the century. The region of the Great Lakes (Malawi and Tanganyika) concentrates the most dramatic increases in extreme event intensities^12^, together with a decrease in the number of rainy days reproduced by most models (Fig. 1d–g). There, the characteristics of daily rainfall will be deeply modified by climate change, should the most pessimistic emission scenarios become reality.

Figure 2e shows to what extent these changes could occur abruptly or, in contrast, gradually. The long-term evolutions of p99 are calculated over a 30-year long moving window over the whole 21st century since 2006. Sampling uncertainties are of sensibly smaller amplitude than the long-term evolution of p99 values (Fig. 2e), a result also verified for lower percentile values. This suggests that these changes in heavy rainfall form a robust feature of climate change over tropical Southern Africa. Of course, from one model to another, the absolute values of p99 strongly vary, denoting large differences in the simulated rainfall (Supplementary Figure 1). For instance, over the region encompassing Lake Tanganyika, p99 fluctuates between 15 mm at the beginning of the simulations (for models HadES and HadAO) to more than 37 mm (according to BCC). For comparison purposes, these values are close to 20 mm in the Twentieth Century Reanalyses^13^ (Supp lementary Figure 2). The amplitude of the changes simulated by the end of the century, and their sensitivity to greenhouse gas concentrations, also exhibit marked differences between the climate models. Yet, none of the models simulate a decrease in the p99 between 2006 and 2100, and all climate models, except HadES for the Tanganyika sector, simulate larger p99 values under RCP8.5 compared to RCP2.6. Among the models considered, five (BNU, CSIRO, FGOALS, HadES, NorESM) show either weak changes or sensitivity to the emission scenarios. Five simulate large increase, strongly determined by the climate change scenario (BCC, CanESM, CCSM, IPSL, MPI). Interpretations are more ambiguous for the third group, because the changes in p99 concern only one region out of the two considered (CNRM, GFDL, MIROC, MRI). The gradual or abrupt nature of the projected increase in p99 values also varies from one model to another, as illustrated on the one hand by models CanESM or IPSL, simulating a continuous and nearly monotonic increase, and on the other hand by BCC, producing more abrupt modifications over both sectors. Taken together, these results suggest that, while the intensification of extreme events is a robust feature of climate change over Southern Africa which is reproduced by all models and can therefore be considered as “very likely”^2^,^8^,^10^,^14^, the amplitude of such increase and the dynamics of its evolution remain much more uncertain, given our current understanding of the climate system.

**Figure 3:**
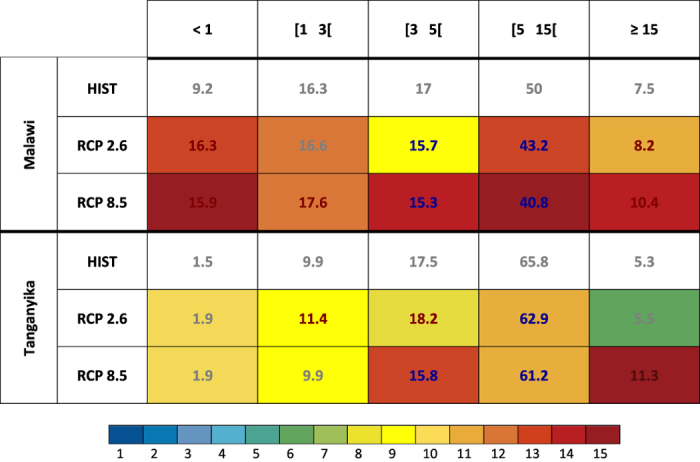
Evolutions in the relative frequency of each category of rainy days, sorted by daily rainfall amounts (mm), for the regional indices Malawi and Tanganyika defined in Fig. 2, and for historical runs (period 1970–1999), and RCP2.6 and 8.5 for period 2070–2099. For RCP simulations, red (blue) text color denotes 95% significant increased (decreased) values against historical runs, grey text color indicates no significant changes. Cell colors indicate the number of models in agreement with the corresponding evolutions and significance, see color bar for legend.

Figure 3 generalizes the analysis of rainfall evolutions over the Malawi and Tanganyika sectors defined in Fig. 2, for the whole distribution of daily rain totals, and not only the rainy days and the extreme events. Given that the climate models still simulate biased distributions of daily rainfall amounts, especially in the tropics, we compare here the structure of rainfall in the historical simulations (HIST, representative of the recent decades under observed concentrations of greenhouse gases) and the future distributions obtained from the emission scenarios RCP2.6 and RCP8.5. Attention is paid to the agreement between climate models, to quantify the probability of occurrence of each simulated evolution.

In the Malawi sector, all models simulate a strong increase in the number of dry days (<1 mm day^−1^), balanced by an increase in the number of extreme rainfall events (>15 mm day^−1^). The frequency of rainy days associated with weak to moderate rainfall amounts, favorable for agriculture, slightly decreases. These evolutions result in an overall stability in the seasonal rainfall amounts (Fig. 1d–f). The consistency between the models is very high (14 to 15 models out of 15) over this region for most rainfall categories, including dry and heavy rainfall days. Even though the long-term evolution of summer rainfall suggest weak impacts for rain-fed agriculture, their decomposition into distinct categories of rainy days leads to radically different conclusions, large daily amounts leading to soil erosion and nutriment leaching while dry sequences may cause crop losses^15^. These detrimental evolutions could be mitigated by lower anthropogenic emissions, as illustrated by the results of RCP2.6 simulations.

In the Tanganyika region, changes in rainfall distribution are more sensitive to the greenhouse gas emission scenario. There is a significant increase in heavy rainfall days associated with RCP8.5 (Fig. 3), while other rainfall categories exhibit weaker, yet significant, changes. Results are also highly reproducible by most climate models. The changes there lead to wetter conditions by the end of the century, suggesting rather favorable conditions for rain-fed agriculture. Yet, this increase occurring mostly during a limited number of intense events, associated impacts for local societies could be weaker than expected, or even detrimental for agronomic yields.

## Superposing long-term circulation changes and short-term anomalies

**Figure 4:**
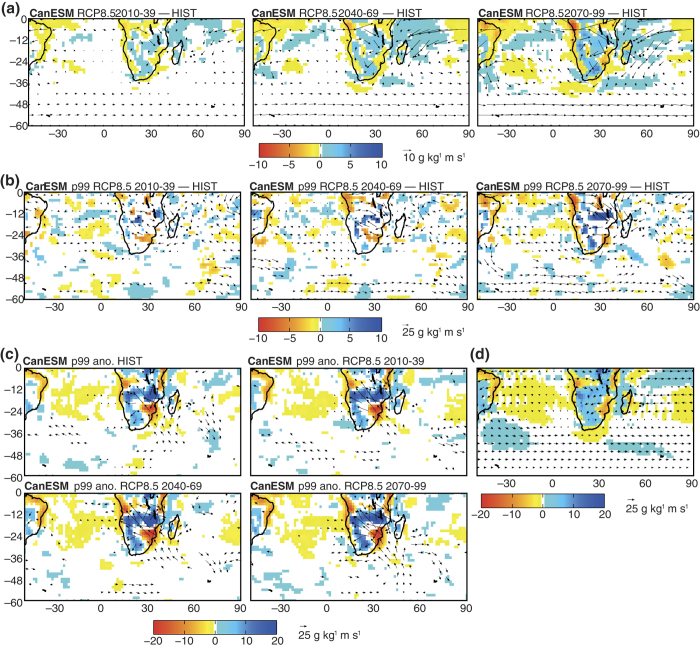
Long-term and short-term changes in moisture fluxes (g kg^−1^ m s^−1^) and moisture convergence (g kg^−1^ s^−1^) in austral summer and during extreme events. (**a**) Long-term evolutions (RCP8.5 minus HIST) of the NDJF seasonal mean moisture fluxes (g kg^−1^ m s^−1^) at 850 hPa, for the CanESM climate model: 2010–2039 (left-hand column), 2040–2069 (central column) and 2070–2099 (right-hand column). Vectors represent 95% significant flux differences and colors represent 95% significant differences in moisture convergence, see color bar and legend. (**b**) As Figure (**a**) but only during the p99 events in the Malawi region (difference between the moisture flux and convergence associated with future p99 events taken from an RCP8.5 simulation minus present-day p99 events obtained from a historical simulation). (**c**) Moisture flux and convergence anomalies during p99 events with respect to their corresponding 30-yr climatology for the same model, and for simulations HIST (period 1970–99) and RCP8.5 (periods 2010–2039, 2040–2069 and 2070–2099). (**d**) Seasonal mean climatology, HIST simulation, period 1970–1999.

The physical causes of the changes described above are investigated in Fig. 4. Here, the CanESM model is chosen as an example for process analysis. More exhaustive discussions considering the diversity of the climate models can be found in the Supplementary Materials. Long-term circulation changes in the region (Fig. 4a) against present-day climate (Fig. 4d) include an intensification of the mid-latitude westerlies south of Africa, pursuing the trend recently observed in the Southern Annular Mode due to stratospheric ozone depletion^6^; such trend is much weaker in the most optimistic emission scenarios^16^. In the tropics, stronger easterly fluxes are found over the tropical Indian Ocean, hereby presenting anomaly patterns reminiscent of the positive phase of the Indian Ocean Dipole^17^. These long-term evolutions are well reproducible by most climate models (Supplementary Figure 3). In addition, the CanESM model simulates westerly anomalies over the equatorial Atlantic, thus promoting zonal moisture convergence over tropical Africa. Even though all models do not simulate such changes in the Atlantic sector, they all reproduce increased moisture convergence over the continent (Supplementary Figure 3). As most continental areas, tropical Africa is a sink for atmospheric moisture: the changes simulated by the end of the century roughly resemble an intensification of the hydrological cycle^9^, enhancing the contrasts between humid and dry zones especially in the tropics (a result summarized by the statement “rich get richer and the poor get poorer”^18^).

During the p99 events over the Malawi sector more specifically, similar long-term changes are noted, although the spatial patterns are noisier due to smaller sample sizes (Fig. 4b). Stronger moisture convergence prevails over tropical Southern Africa during future extreme rainfall events. This suggests that the average changes discussed above will be exaggerated during heavy rainfall events. Figure 4c further investigates this issue. Instead of computing the differences between future and present p99 events (as in Fig. 4b), we analyze here the short-lived anomalies against their corresponding 30-year climatology (that is, the climate mean state for the early, mid- or late century). From one period to another, including both historical and RCP8.5 simulations, short-term moisture flux and convergence anomalies during heavy rainfall days are remarkably stable (Fig. 4c). Lower-layer fluxes converge over tropical Africa and convey precipitable there, originating from both Atlantic and Indian Ocean basins. Southeasterly anomalies over the south-west Indian Ocean denote an abnormally weak South Indian Convergence Zone^19^ during these days. This promotes meridional convergence over the subcontinent. Moisture divergence anomalies prevail on both sides of the Great Lakes, on the South Atlantic and along the east coast of Southern Africa. The stationarity of these features indicate that the increase in the p99 values (Fig. 2) results from the superposition of short-lived (synoptic-scale) circulation anomalies, which are mostly unmodified from the late 20th century until the end of the 21st century, and slowly-varying long-term changes directly driven by anthropogenic greenhouse gas emissions. The latter are therefore strongly sensitive to the emission scenarios for future decades, the most optimistic scenario RCP2.6 corresponding to the lowest increase in the extreme event intensities (Figs 2e and 3).

Additional analyses help separate the effects of wind and air moisture content. Indeed, warmer air masses are expected to content increased moisture and precipitable water, in line with the Clausius-Clapeyron relation. Even though both influences are involved (Supplementary Figure 6), enhanced specific humidity is responsible for the long-term increase in moisture convergence over tropical Southern Africa, the latter promoting the intensification of rainfall extremes. This is why optimistic scenarios, by limiting air mass warming, also produce weaker changes in the p99 values. All of these conclusions also hold for the Tanganyika region (Supplementary Figure 7).

These results show that the climate models are much more consistent for projecting changes in the structure of daily rainfall, namely more intense extreme events and fewer rainfall days^2^,^10^, than for seasonal totals. More precise and detailed results could be obtained from high-resolution limited area models, shown to outperform global models, especially for simulating intense rainfall events^20^. These changes simulated over Southern Africa may sensibly alter the quality of the future rainy seasons, from an agronomic perspective. More frequent dry days or more persistent dry sequences may cause strong decrease in agronomic yields, if occurring during key phases of the phenology (e.g., the flowering phase) when the plant is vulnerable^15^. Abundant rainfall amounts may also reduce soil fertility due to nitrogen leaching. Although this could add uncertainties and make the modeling chain more complex, inter-comparing crop-models over regions experiencing such evolutions in their daily rainfall (see for instance http://www.agmip.org/)^21^ could allow separating the impacts of climate change from the errors and uncertainties associated with both agronomic and climate models. This could help anticipating such changes and adopt relevant mitigation strategies.

## Methods

All differences and anomalies presented and discussed in this work are significant at the 95% level according to statistical tests assessing whether the means of two samples are different from each other. For scalar fields (seasonal rainfall amounts, frequency and intensity of rainy days in Fig. 1, intensity of p99 events in Fig. 2, frequency of rainy day categories in Fig. 3 and moisture convergence in Fig. 4), we used a two-tailed Student *t*-test. For vector fields (moisture fluxes in Fig. 4) we used Hotelling’s *t*^*2*^-test, which is the multivariate generalization of the *t*-test. Here, it is applied to the zonal and meridional components of the fluxes.

## Additional Information

**How to cite this article:** Pohl, B. *et al*. Fewer rainy days and more extreme rainfall by the end of the century in Southern Africa. *Sci. Rep.* **7**, 46466; doi: 10.1038/srep46466 (2017).

**Publisher's note:** Springer Nature remains neutral with regard to jurisdictional claims in published maps and institutional affiliations.

## Supplementary Material

Supplementary Information
