# Supplementary material for: Fewer rainy days and more extreme rainfall by the end of the century in Southern Africa
Source: Sci Rep. 2017 Apr 13;7:46466. doi: 10.1038/srep46466 (PMC5390289; doi:10.1038/srep46466)
Supplement: Supplementary Information [file srep46466-s1.doc]

**Fewer rainy days and more extreme rainfall**

**by the end of the century in Southern Africa**

Benjamin Pohl, Clémence Macron

Paul-Arthur Monerie^b^

**Supplementary Information**

**Supplementary Methods**

In this paper we selected 15 model versions out of the 39 reported in the fifth Assessment Report of IPCC^1^. This choice is motivated by various factors: (i) we tried to adopt the well-known "one model, one vote"^2^ approach to give the same weight to each model unless two versions lead to dissimilar results, which is not the usual case^3,4^; (ii) we tried to respect an “institutional democracy”^3^; (iii) we selected all the models for which the requested simulations (historical, RCP2.6 and RCP8.5) and variables (rainfall amounts, water vapor mixing ratio, horizontal wind components) were archived at the daily timescale and accessible online. Supplementary Table 1 summarizes the model versions used here, together with the institutions in charge of the model developments and the acronyms used in this work. More detailed information concerning these models can be found in the fifth Assessment Report of IPCC^1^ (Working Group I, Chapter 9 ).

| **Models** | **Institute (Country)** | **Acronym** |
| --- | --- | --- |
| BCC-CSM1.1 | Beijing Climate Center (China) | BCC |
| BNU-ESM | Beijing Normal University (China) | BNU |
| CanESM2 | Canadian Centre for Climate Modelling and Analysis, Canada | CanESM |
| CCSM4 | National Center for Atmospheric Research (USA) | CCSM |
| CNRM-CM5 | Centre National de Recherches Météorologiques / Centre Européen de Recherche et Formation Avancées en Calcul Scientifique (France) | CNRM |
| CSIRO-Mk3.6.0 | Commonwealth Scientific and Industrial Research Organisation (Australia) | CSIRO |
| FGOALS-g2 | Institute of Atmospheric Physics, Chinese Academy of Sciences and Tsinghua University (China) | FGOALS |
| GFDL-ESM2M | Geophysical Fluid Dynamics Laboratory (USA) | GFDL |
| HadGEM2-AO | National Institute of Meteorological Research, Korea Meteorological Administration (Korea) | HadAO |
| HadGEM2-ES | Met Office Hadley Centre (UK) | HadES |
| IPSL-CM5A-LR | Institut Pierre-Simon Laplace (France) | IPSL |
| MIROC-ESM | Atmosphere and Ocean Research Institute / National Institute for Environmental Studies, and Japan Agency for Marine-Earth Science and Technology (Japan) | MIROC |
| MPI-ESM-MR | Max Planck Institute for Meteorology (Germany) | MPI |
| MRI-CGCM3 | Meteorological Research Institute (Japan) | MRI |
| NorESM1-M | Norwegian Climate Centre (Norway) | NorESM |

**Supplementary Table 1 |** Summary of the model versions (such as they appear in the fifth Assessment Report of IPCC, left-hand column), associated institutions and countries (central column) and acronym retained for this work (right-hand column).

In the main paper, special attention is given to the degree of confidence of the simulated changes in rainfall days and extreme event intensities, as well as the physical causes of these changes. This is quantified through the number of climate models, out of the 15 presented in Supplementary Table 1, that simulate changes that are in agreement with the multi-model average. By agreement we mean change of the same sign (i.e., increase or decrease of a given variable), but also with the same statistical significance in the simulated evolution. The latter is estimated by comparing future climate conditions obtained from experiments forced by RCP2.6 and RCP8.5 radiative forcings, against historical simulations of present-day climate, the difference between both simulations and periods being tested statistically at the 95% confidence bound by a two-tailed Student *t*-test for scalar fields, and *t^2^*-test (also known as Hotelling's test) for vector fields. The *t^2^*-test is the multivariate generalization of the *t*-test. In this study it is applied to the zonal and meridional components of the horizontal moisture or mass fluxes.

**Supplementary Discussion and Figures**

**1. Rainfall biases of the CMIP5 climate models**

All analyses presented in the main paper were performed for all the models presented in Supplementary Table 1. This is necessary to ensure that the results discussed and presented here are robust, to assess their model-dependency, and finally to quantify the number of models simulating convergent trends or changes. Indeed, a major issue addressed in this study concerns the consensus between the climate models, much larger for the increase in extreme rainfall intensities over Southern Africa than for seasonal amount evolutions. Below, we propose to further document the resemblance of the models, through detailed analyses of their simulated changes, and associated mechanisms. For conciseness we present hereafter the results from 8 models only, representative of the whole ensemble. This allows us proposing more detailed analyses and discussions about the specificities of each model.

Supplementary Figure 1 shows the biases of the models in terms of seasonal mean rainfall during the austral summer season over Southern Africa, as well as the number of rainfall days and the average amounts of rainy days. ERA40 reanalyses were selected as a reference dataset because it is available at the daily timescale over the whole period 1970-1999 analyzed in this work. The quality and deficiencies of the rainfall climatology provided by this dataset over the Southern Africa region have already been discussed in detail in previous work^5–8^: the differences from one model to another remain of first order compared to the uncertainties coming from the rainfall estimations at large scales (as shown by inter-comparisons between interpolated rain-gauge records, reanalyses and satellite estimates^9,10^). Even if reanalyses are not perfect, especially in the tropics^11^ and more particularly Africa^12^, they remain sensibly closer to remote-sensing estimates or in situ measurements than current climate models against all these pseudo-observations (and, most of the time, than climate models between them).

According to ERA40 (and various measurement-based or satellite rainfall estimates, not shown), largest rainfall amounts over the domain are confined north of 15°S, including Southern Africa and the central Indian Ocean. There, average daily amounts typically exceed 5-10 mm (Supplementary Figure 1a). One noticeable exception is found at the longitudes of Madagascar, the Mozambique Chanel and the nearby southwest Indian Ocean. There, a northwest-southeast oriented band links the southern mid-latitudes to the tropics and is known as the South Indian Convergence Zone^13^. Drier conditions (daily rainfall exceeding barely 2 mm) are found on each side, due to the prevalence of dynamical subtropical subsidence, over the Benguela region off the west coast of Southern Africa, and over the eastern Indian Ocean west of Australia.

**Supplementary Figure 1 |** Rainfall biases of a sample of 8 CMIP5 models. (a) Seasonal mean rainfall amounts. Left-hand panel: seasonal mean rainfall (mm day^-1^) averaged during the austral summer season (November through February) according to ERA40^14^, period 1970-1999. Right-hand panels: Average seasonal biases (mm day^-1^) of a sample of 8 CMIP5 models, historical simulations, over the same period as above and against ERA40 reanalyses. See color bars for color signification and Supplementary Table 1 for model acronyms. (b) As (a) but for the number of rainy days (> 1 mm total daily amount). (c) As (a) but for the average intensity of rainy days (mm). [Figure made by the authors with Matlab]

This geography of the rainfall field is very similar with that of the number of rainy days (Supplementary Figure 1b), except in the southern mid-latitudes characterized by moderate total rainfall amounts in spite of a large number of rainy days, the latter being on average associated with small amounts (lesser than 3-4 mm, against 10 and more in some tropical regions, see Supplementary Figure 1c). Over Southern Africa, most of the spatial contrasts found in the mean rainfall field actually result from the number of rainy days, their average intensities being much mode constant spatially.

Compared to this mean state, CMIP5 climate models present biases that cannot be considered as negligible. They locally reach up to 6 mm day^-1^ (about 50% of the largest daily amounts), 40 rainy days (one third of the length of the rainy season, from November through February) and about 10 mm for rainy day intensities, two thirds of the largest values found over the domain.

From one model to another the biases strongly vary. All models tend to produce too abundant rainfall over the tropical Indian Ocean and too dry conditions over the southeast Atlantic basin, and to a lesser extent, over the subtropical Indian Ocean (Supplementary Figure 1a). In the mid-latitudes, and even more clearly over Southern Africa, more contrasted situations are obtained, the sign of the biases changing between the models.

All models also tend to produce too many rainy days (Supplementary Figure 1b), a result well known in the tropics, except over the southeast Atlantic over the upwelling region of Benguela. Some of the models also underestimate the frequency of rainfall over Madagascar (this is the case for the BCC model) or the subtropical Indian Ocean east of is (models CanESM, MPI and NorESM). Except for the Benguela region, errors are more model-dependent for the intensity of rainy days (Supplementary Figure 1c), biases of both signs being found over Southern Africa, the Indian Ocean and the mid-latitudes.

Do these biases alter the long-term changes in the rainfall distributions commented in the main paper? There is no possibility to answer this question unambiguously. A very large number of studies using the CMIP models use the so-called delta-change approach, assuming that the errors are constant in time and thus do not modify the changes simulated between future decades and present-day climate conditions. This is also the method adopted in this work.

**2. Recent evolutions in extreme rainfall over Southern Africa**

The main paper suggests that Southern Africa, and more particularly the Malawi and Tanganyika regions, may experience future evolutions in their daily rainfall, which consist in fewer rainy days and more intense heavy rainfall events. Given that the influence of human activities on the climate is significant since at least the 1960s^1^, such changes could already be discernible in the structure of African rainfall in recent observations. The difficulty is that long-term observational time series available at the daily timescale are excessively rare over Southern Africa. This is even more true at the tropical latitudes, where climate models simulate the largest changes by the end of the century. The longest observational records available over the region are those from South Africa, where future evolutions of daily rainfall are of weak amplitude (Figs. 1 and 2).

Satellite estimates of daily rainfall only exist since 1996 and the availability of active (microwave) sensors^15^: this period is too short to properly extract robust low-frequency changes. Atmospheric reanalyses (discussed in Section 1) could be another potential candidate to reconstruct recent climate variability. However, strong difficulties arise when working on long-term changes, most of which relate to the temporal inconsistencies in the amounts, types and quality of observational databases used for the assimilation, shown to lead to spurious long-term trends^16,17^. Over Africa, where in situ measurements and radiosonde data are very rare, the introduction of satellite data in the late 1970s lead to significant breaks in the reanalyses^12^, which make it difficult to disentangle methodological artifacts and low-frequency climate signals (such as, for instance, the change of sign of the Pacific Decadal Oscillation in 1977-78^18–20^). Moreover, the quality of the reanalyses is often uncertain in the tropics^11^, especially for the variables which are not directly constrained by the assimilation, and are more dependent on the model physics^21^. This is for instance the case for (stratiform and convective) precipitation totals. Therefore, their interpretation requires extreme caution^11,12^.

One possible compromise to assess long-term evolutions (over a few decades) in heavy daily rainfall over regions where there are no observational records is to use the recent 56-member ensemble Twentieth Century Reanalyses^22^ version 2c (20CR hereafter). 20CR assimilate no satellite data or radiosonde to avoid such artificial ruptures, and are thus more appropriate for the analysis of low-frequency variability. Although the type of assimilated data (namely, surface pressure measurements) remains constant since the late 19^th^ century, the density of the network shows marked fluctuations during the 20^th^ century, and the quality of the monthly sea surface temperature and sea-ice distributions used as boundary conditions also shows sensible improvements over the period. Over Africa in particular, the density of the International Surface Pressure Databank observational network, assimilated in the 20CR, abruptly increases in 1967. This could induce significant ruptures in the reanalyzed fields. For the present work, we chose therefore to restrain our analyses to the period 1967-2011, for which the amounts of assimilated data remains more or less constant, even if it still varies from one year to another.

Results for extreme rainfall events are shown in Supplementary Figure 2. Supp. Fig. 2a shows a general increase in the daily p99 values between the sub-periods 1967-88 and 1989-2011, even if localized sectors near Madagascar are characterized by a strong decrease. The geography of the recent changes (Supp. Fig. 2a) slightly differs from the projected changes for future decades (Fig. 2a-d), the largest evolutions being found over Botswana and eastern Namibia instead of the Malawi and Tanganyika sectors further North. Once again, caution must be used when interpreting these signals in the 20CR. Precipitation amounts in the tropics, and extreme precipitations in particular, are among the most difficult variables to simulate for current atmospheric models, with or without assimilation.

Over the key regions identified in the main paper, daily p99 computed over a 30-year moving window also display marked increase (Supp. Fig. 2b-c), statistically significant at the 99% confidence level. This evolution is reproduced by all of the individual members: even though the absolute p99 values vary from one member to another, all of them show similar magnitudes in the long-term changes (of about +1 mm day^-1^). This is sensibly weaker than the changes projected for the 21^st^ century (Fig. 2), especially under the RCP8.5 radiative forcing, which could denote an increasing human influence on climate. Given the relative shortness of the period considered here, and the edge effects associated with the 30-year long moving window used to compute the daily p99 values, Supp. Fig. 2d-e present the largest daily precipitation amounts recorded each year over the Malawi and Tanganyika regions. Associated time series are much noisier, and more uncertain within the ensemble. In line with strong interannual variability, long-term trends are not statistically significant at the 95% level. Yet, recent years tend to show larger daily maximum values than the early decades of the period, suggesting possible, but uncertain, intensifications in extreme rainy days.

Longer time series, ideally derived from direct in situ measurements, are needed to properly assess these changes.

**Supplementary Figure 2 |** Recent evolutions in heavy precipitation over Southern Africa according to 20CR. (a) Difference between the 56-member ensemble mean of p99 daily rainfall (mm) in NDJF 1989-2011 minus NDJF 1967-1988. Only significant differences according to a two-tailed t-test at the 95% confidence level are displayed. The dashed rectangles show the Malawi and Tanganyika indices, as used in the main paper. (b) Temporal evolution (period NDJF 1967-2011) of the p99 values computed over a 30-year long moving window over the Malawi region. The years indicated in the x-axis correspond to the first year of the moving window. The 56 individual members of 20CR appear as cyan curves and their ensemble mean is shown by the red curve. (c) As (b) but for the Tanganyika region. (d) As (b) but for the largest daily rainfall amount of each year, period NDJF 1967-2011. (e) As (d) but for the Tanganyika region. [Figure made by the authors with Matlab]

**3. Long-term circulation changes vs. short-term anomalies during extreme events**

The main paper concludes on the superposition of slowly-changing circulation patterns and stationary short-term anomalies to explain the increase in the 99^th^ percentile value of daily rainfall amounts, the long-term changes favouring increased moisture convergence over Southern Africa. Additional analyses (not shown) reveal (i) that qualitatively similar results and similar conclusions can be obtained when considering the 90^th^, 95^th^ or 99^th^ percentiles, the weaker percentiles leading to the smoother fields and the larger resemblance between extreme events and seasonal mean fields due to larger sample sizes; (ii) that computing these percentiles on the whole time series or only on rainy days does not change the results, the CMIP5 models tending to produce too many rainy days that occupy almost 90 to 100% of the austral summer rainy season over Africa (Supplementary Figure 1b).

The results presented in the main paper are based on one single climate model, namely CanESM, used for illustration. This section explores the differences between the models, to quantify the uncertainties of these evolutions and therefore the robustness of these conclusions.

Supplementary Figure 3 shows the long-term circulation changes simulated by the same sample of 8 climate models over the 21st century. All models simulate a strong increase in the mid-latitude westerly moisture transport, gradually increasing in time: this feature of climate change in the Southern Hemisphere has already attracted a large number of publications. All models also produce increased moisture fluxes from the Indian Ocean basin towards Southern Africa, the main differences concerning the latitude at which the lower-layer fluxes reach and penetrate over the subcontinent. CanESM (discussed in the main paper) but also GFDL, MIROC and MPI place these easterly anomalies, interpretable as an enhancement of the trade winds, in the tropics. BNU, CNRM and NorESM place them further south, at the subtropical latitudes. BCC produces more widespread changes, that concern almost the whole eastern coast of Southern Africa.

In the South Atlantic sector models also tend to simulate an enhancement of the trade winds, albeit of different magnitude and location. The strongest (weakest) increase is produced by BNU (CNRM and MIROC). Concerning Southern Africa, the models tend to simulate a weak decrease in moisture transport from the continent towards the tropical Atlantic Ocean. This takes the form of westerly flux differences developing in future decades compared to the historical simulations. Compared to the increased moisture transport from the Indian Ocean, these changes are of much smaller amplitude and spatial extension. Yet, taken together, these combined modifications promote zonal moisture convergence over Southern Africa, an evolution reproduced by all models.

**Supplementary Figure 3 |** Long-term evolutions (RCP8.5 minus HIST) of the NDJF seasonal mean moisture fluxes (g kg-1 m s-1) at 850hPa, for a sample of 8 climate models (rows; CanESM omitted since presented in the main paper): 2010-2039 (left-hand column), 2040-2069 (central column) and 2070-2099 (right-hand column). Vectors represent 95% significant flux differences and colors represent 95% significant differences in moisture convergence, see color bar and legend. [Figure made by the authors with Matlab]

**Supplementary Figure 4 |** As Supplementary Figure 3 but only during the p99 events in the Malawi region (difference between the moisture flux and convergence associated with future p99 events taken from an RCP8.5 simulation minus present-day p99 events obtained from a historical simulation). [Figure made by the authors with Matlab]

Like Figure 4b of the main paper, Supplementary Figure 4 shows the same long-term evolutions but only during extreme rainfall (p99) events over the Malawi sector, instead of seasonal means. As stated in the main paper, one can find here the most robust features of climate change that already appear on seasonal mean fields, even though the signals are much noisier due to the reduced sample sizes. Strong moisture convergence anomalies prevail near the Great Lake region, over tropical Southern Africa: the fluxes originate from both adjacent oceans, but their magnitude and the relative weight of both origins vary between models. Once again the anomaly patterns found during extreme events in the tropics closely resemble the seasonal mean long-term changes simulated by each model, and shown for our sample of 8 models in Supplementary Figure 3. For instance, the models producing an enhancement of the trade winds over the tropical Indian Ocean simulate moisture fluxes synchronous to extreme events that gradually originate more predominantly from the Indian basin; for models producing also westerly differences over the tropical Atlantic, moisture convergence over Southern Africa results from both basins together, and is enhanced because of these combined modifications. One can thus question the timescale at which these changes occur: from one decade to another, or specifically during heavy or extreme rainfall events, the latter contributing thus primarily to the mean long-term circulation changes. In order to address this issue, and as achieved in the main paper for one climate model only, we propose here to separate the long-term changes and the short-term anomalies simulated concomitantly with extreme rainfall events: short-term synchronous anomalies during p99 events against their corresponding 30-year mean climatology are shown in Supplementary Figure 5.

The circulation anomalies associated with p99 events over the Malawi region sensibly vary from one model to another (Supplementary Figure 5). Logically, they all correspond to local lower-tropospheric moisture convergence over tropical Southern Africa. Synchronous convergence anomalies are strong over most parts of the subcontinent, and especially over the East African Rift and nearby reliefs. This pattern is reproduced by all models. Moisture fluxes reach tropical Southern Africa from both the equatorial Atlantic and Indian Oceans. Weaker moisture transport occur over the southwest Indian Ocean, which can be interpreted as an abnormally weak South Indian Convergence Zone^13^: this feature is among those for which there is the largest consensus between the models (with two exceptions, MIROC and GFDL). Another common and well reproducible feature lies in the tendency for all models to produce large-scale divergent anomalies over both ocean basins at the southern tropical latitudes. However, over these regions, moisture flux anomalies are both weaker and less consistent between models. Strong differences are found in their directions, as well as their magnitude.

Yet, for all models, the short-term anomaly patterns that occur synchronously with p99 events are remarkably stable from one simulation or period to another. The conclusions stressed in the main paper and based on one single climate model hold for the sample of 8 models presented here, and even for the 15 models retained for this study (not shown). The physical causes for the increase in the p99 values are the superposition of stationary short-term anomalies to a slowly changing circulation mean state. While the short-term anomalies are unchanged between historical simulations, and those forced by RCP2.6 and RCP8.5 greenhouse gas concentrations, the long-term circulation changes are much larger in the RCP8.5 simulations than in their RCP2.6 counterparts, hereby denoting a primary influence of human societies in the simulated future climate changes.

**Supplementary Figure 5 |** Moisture flux at 850hPa (vectors) and moisture convergence anomalies (colors) during p99 events with respect to their corresponding 30-yr climatology for the same sample of 8 models, and for simulations HIST (period 1970-99) and RCP8.5 (periods 2010-2039, 2040-2069 and 2070-2099). [Figure made by the authors with Matlab]

*(Continued)*

**4. Changes in lower-layer wind vs. air humidity**

Additional analyses based on mass fluxes instead of moisture fluxes aim at determining to what extent the moisture flux changes discussed above can be attributed to the atmospheric dynamics (that is, in the large-scale horizontal wind in the lower troposphere) or to the moisture content of the air masses. They are shown in Supplementary Figure 6.

Comparisons with Figure 4 reveal the following salient results:

— Long-term changes in the seasonal fluxes are weaker for mass fluxes than for moisture fluxes. This is for instance the case over Southern Africa: the gradual increase in flux convergence over the continent is not so evident in the lower-layer wind. Another example comes from the southern mid-latitudes: while moisture fluxes give the picture of a generalized enhancement of the westerlies south of 40°S (the "roaring forties"), analysis of the wind changes reveal more a latitudinal shift towards the pole, the westerly fluxes decreasing between 35°S and 50°S and increasing further south. The differences between the two figures come from air moisture content (also known as specific humidity, and expressed here in g of water vapour per kg of air), the latter being increased with global warming due to larger hygrometric capacity. This is a direct consequence of the Clausius-Clapeyron relation. Thus, increased moisture convergence over Southern Africa results from both changes in the lower-layer wind (especially over the near-equatorial Indian and Atlantic Oceans) but also in specific humidity, promoting stronger moisture transport and convergence.

— The differences between both figures are even more evident for moisture flux and convergence differences during extreme rainfall events (Fig. 4b and Supp. Fig. 6b). In particular, the strong increase in moisture convergence over tropical Africa during extreme rainfall events is not accompanied by such a counterpart in mass fluxes: the amplitude of the synchronous anomalies in the wind show a much more limited increase throughout the century. This highlights once again the predominant role of air moisture content.

— As for moisture fluxes, short-term anomalies (with respect to their respective climatologies) during extreme events remain stationary from one simulation or period to another (Fig. 4c and Supp. Fig. 6c). This shows that the structure of short-lived atmospheric patterns in which the rainfall extreme events are embedded is unmodified by climate change over the century.

Thus, the increase in extreme event intensities is caused by the superposition of stationary short-term configurations, and slowly changing mean circulation. The latter involve both changes in the atmospheric dynamics and increased moisture transport due to enhanced hygrometric capacity.

**Supplementary Figure 6 |** As Figure 4 of the main paper but for mass fluxes at 850 hPa (m s^-1^) instead of moisture fluxes. [Figure made by the authors with Matlab]

**5. Scale superpositions for the Tanganyika region**

Supplementary Figure 7 generalizes the analyses performed over the Malawi sector (in the main paper and sections 2 and 3 above) over the Tanganyika region. The main conclusions concerning the complementary effects of long-term changes in the mean atmospheric circulation and unmodified short-lived anomaly patterns around an evolving climatology are also verified for this second regional index. As for the Malawi sector, the intensification of heavy rainfall events over and around Lake Tanganyika result from the superposition, that is, the additive effects, of low-frequency evolutions and barely modified synoptic-scale perturbations at higher frequencies. This suggests that these mechanisms could account for most of the long-term changes noted in this study in the structure of daily rainfall distributions.

**Supplementary Figure 7 |** As Figure 4b,c of the main paper but for the Tanganyika region (shown in Fig. 2). [Figure made by the authors with Matlab]

**6. Perspectives**

From a statistical point of view, the methodology used to extract the short-term circulation anomalies in which sub-regional extreme events are embedded remains very simple. Additional work is now required to better document the variety of such anomalies, and the modes of natural climate variability (e.g., El Niño Southern Oscillation^23–26^ or the Indian Ocean Dipole^27^) that could favour them. This could be done for instance by using a decomposition into recurrent weather or circulation regimes^28,29^.

From a model point of view, the use of general circulation models allows for the analysis of teleconnections between a given region of interest (e.g., the Malawi or Tanganyika sectors) and remote regions (the adjacent ocean basins in our case). The main limitation concerns the moderate spatial resolution (usually, a few tens of kilometers in the CMIP5 exercise) and the perfectible distribution of daily rainfall (especially the strong overestimation of the number of rainy days), a statement particularly true in the tropics where convective rainfall predominates (Supplementary Figure 3). These limitations could be partly overcome by using limited area models, also known as regional climate models^30,31^, which only simulate the climate over the region of interest (e.g., Africa and its surroundings) and can reach higher spatial resolution at reasonable computational costs. The benefits in terms of tropical rainfall distributions have been shown to be significant, a conclusion especially verified for extreme events^32,33^. The main limitation of these models involve spatial scale interactions, that is, the local influence of large-scale climate variability^6,23^ or the lack of feedback effects of the local / regional climate variability onto the global circulation. Future work could also attempt to quantify the added-value versus the uncertainties associated with regional climate models for the analysis of the (present-day and future) heavy rainfall events over Southern Africa^34,35^.

**Additional references**

1. Intergovernmental Panel on Climate Change. *Climate Change 2013: The Physical Science Basis. Contribution of Working Group I to the Fifth Assessment Report of the Intergovernmental Panel on Climate Change*. (2013).

2. Knutti, R. The end of model democracy? *Clim. Change* **102,** 395–404 (2010).

3. Leduc, M., Laprise, R., de Elía, R. & Šeparović, L. Is institutional democracy a good proxy for model independence? *J. Clim.* **in press,** (2016).

4. Monerie, P.-A., Sanchez-Gomez, E. & Boé, J. On the range of future Sahel precipitation projections and the selection of a sub-sample of CMIP5 models for impact studies. *Clim. Dyn.* (2016). doi:10.1007/s00382-016-3236-y

5. Vigaud, N., Pohl, B. & Crétat, J. Tropical-temperate interactions over southern Africa simulated by a regional climate model. *Clim. Dyn.* **39,** 2895–2916 (2012).

6. Boulard, D., Pohl, B., Crétat, J., Vigaud, N. & Pham-Xuan, T. Downscaling large-scale climate variability using a regional climate model: the case of ENSO over Southern Africa. *Clim. Dyn.* **40,** 1141–1168 (2013).

7. Crétat, J., Pohl, B., Vigaud, N. & Richard, Y. An original way to evaluate daily rainfall variability simulated by a regional climate model: the case of South African austral summer rainfall. *Int. J. Climatol.* **35,** 2485–2502 (2014).

8. Crétat, J. *et al.* Recurrent daily rainfall patterns over South Africa and associated dynamics during the core of the austral summer. *Int. J. Climatol.* **32,** 261–273 (2012).

9. Crétat, J. Pluviométrie et circulation atmosphérique simulées par le modèle régional WRF en Afrique australe : sensibilité à la physique et variabilité interne. (Université de Bourgogne, 2011).

10. Macron, C. Les Talwegs Tropicaux Tempérés : dynamique atmosphérique associée et évolution future. (Université de Bourgogne, 2014).

11. Trenberth, K. E., Stepaniak, D. P., Hurrell, J. W. & Fiorino, M. Quality of reanalyses in the tropics. *J. Clim.* **14,** 1499–1510 (2001).

12. Poccard, I., Janicot, S. & Camberlin, P. Comparison of rainfall structures between NCEP/NCAR reanalyses and observed data over tropical Africa. *Clim. Dyn.* **16,** 897–915 (2000).

13. Cook, K. H. The South Indian Convergence Zone and Interannual Rainfall Variability over Southern Africa. *J. Clim.* **13,** 3789–3804 (2000).

14. Uppala, S. M. *et al.* The ERA-40 re-analysis. *Q. J. R. Meteorol. Soc.* **131,** 2961–3012 (2005).

15. Huffman, G. J. *et al.* Global precipitation at one-degree daily resolution from multisatellite observations. *J. Hydrometeorol.* **2,** 36–50 (2001).

16. Sturaro, G. A closer look at the climatological discontinuities present in the NCEP/NCAR reanalysis temperature due to the introduction of satellite data. *Clim. Dyn.* **21,** 309–316 (2003).

17. Kinter, J. L., Fennessy, M. J., Krishnamurthy, V. & Marx, L. An evaluation of the apparent interdecadal shift in the tropical divergent circulation in the NCEP-NCAR reanalysis. *J. Clim.* **17,** 349–361 (2004).

18. Mantua, N. J., Hare, S. R., Zhang, Y., Wallace, J. M. & Francis, R. C. A Pacific interdecadal climate oscillation with impacts on salmon production. *Bull. Am. Meteorol. Soc.* **78,** 1069–1079 (1997).

19. Zhang, Y., Wallace, J. M. & Battisti, D. S. ENSO-like in- terdecadal variability: 1900–93. *J. Clim.* **10,** 1004–1020 (1997).

20. Deser, C., Phillipps, A. S. & Hurrell, J. W. Pacific interdecadal climate variability: Linkages between the Tropics and the North Pacific during boreal winter since 1900. *J. Clim.* **17,** 3109–3124 (2004).

21. Kalnay, E. *et al.* The NCEP/NCAR 40-Year Reanalysis Project. *Bull. Am. Meteorol. Soc.* **77,** 437–471 (1996).

22. Compo, G. P. *et al.* The twentieth century reanalysis project. *Q. J. R. Meteorol. Soc.* **137,** 1–28 (2011).

23. Meque, A. & Abiodun, B. J. Simulating the link between ENSO and summer drought in Southern Africa using regional climate models. *Clim. Dyn.* **44,** 1881–1900 (2014).

24. Reason, C. J. C. & Jagadheesha, D. A model investigation of recent ENSO impacts over southern Africa. *Meteorol. Atmos. Phys.* **89,** 181–205 (2005).

25. Dieppois, B., Rouault, M. & New, M. The impact of ENSO on Southern African rainfall in CMIP5 ocean atmosphere coupled climate models. *Clim. Dyn.* **45,** 2425–2442 (2015).

26. Hoell, A., Funk, C., Magadzire, T., Zinke, J. & Husak, G. El Niño – Southern Oscillation diversity and Southern Africa teleconnections during Austral Summer. *Clim. Dyn.* **45,** 1583–1599 (2015).

27. Saji, N. H., Goswami, B. N., Vinayachandran, P. N. & Yamagata, T. A dipole mode in the tropical Indian Ocean. *Nature* **401,** 360–363 (1999).

28. Cassou, C. Intraseasonal interaction between the Madden–Julian oscillation and the North Atlantic oscillation. *Nature* **455,** 523–527 (2008).

29. Fauchereau, N., Pohl, B., Reason, C. J. C., Rouault, M. & Richard, Y. Recurrent daily OLR patterns in the Southern Africa/Southwest Indian Ocean region, implications for South African rainfall and teleconnections. *Clim. Dyn.* **32,** 575–591 (2009).

30. Giorgi, F. Regional climate modeling: Status and perspectives. *J. Phys. IV* **139,** 101–118 (2006).

31. Laprise, R. Regional climate modelling. *J. Comput. Phys.* **227,** 3641–3666 (2008).

32. Crétat, J., Vizy, E. K. & Cook, K. H. How well are daily intense rainfall events captured by current climate models over Africa? *Clim. Dyn.* **42,** 2691–2711 (2014).

33. Pinto, I. *et al.* Evaluation and projections of extreme precipitation over southern Africa from two CORDEX models. *Clim. Change* **135,** 655–668 (2016).

34. Shongwe, M. E. *et al.* Projected changes in mean and extreme precipitation in Africa under global warming. Part I: Southern Africa. *J. Clim.* **22,** 3819–3837 (2009).

35. Mason, S. J. & Joubert, A. M. Simulated changes in extreme rainfall over southern Africa. *Int. J. Climatol.* **17,** 291–301 (1997).
